# Boundary behaviours of *Leishmania mexicana*: A hydrodynamic simulation study

**DOI:** 10.1016/j.jtbi.2018.11.016

**Published:** 2019-02-07

**Authors:** Benjamin J. Walker, Richard J. Wheeler, Kenta Ishimoto, Eamonn A. Gaffney

**Affiliations:** a Wolfson Centre for Mathematical Biology, Mathematical Institute, University of Oxford, Oxford, OX2 6GG, UK; b Sir William Dunn School of Pathology, University of Oxford, Oxford, OX1 3RE, UK; c Peter Medawar Building for Pathogen Research, Nuffield Department of Medicine, University of Oxford, Oxford, OX1 3SY, UK; d Graduate School of Mathematical Sciences, The University of Tokyo, Tokyo, 153–8914, Japan

**Keywords:** Promastigote motility, Boundary element method, Flagellar beat, Low Reynolds number flow, *Leishmania*-sandfly gut interaction

## Abstract

It is well established that the parasites of the genus *Leishmania* exhibit complex surface interactions with the sandfly vector midgut epithelium, but no prior study has considered the details of their hydrodynamics. Here, the boundary behaviours of motile *Leishmania mexicana* promastigotes are explored in a computational study using the boundary element method, with a model flagellar beating pattern that has been identified from digital videomicroscopy. In particular a simple flagellar kinematics is observed and quantified using image processing and mode identification techniques, suggesting a simple mechanical driver for the *Leishmania* beat. Phase plane analysis and long-time simulation of a range of *Leishmania* swimming scenarios demonstrate an absence of stable boundary motility for an idealised model promastigote, with behaviours ranging from boundary capture to deflection into the bulk both with and without surface forces between the swimmer and the boundary. Indeed, the inclusion of a short-range repulsive surface force results in the deflection of all surface-bound promastigotes, suggesting that the documented surface detachment of infective metacyclic promastigotes may be the result of their particular morphology and simple hydrodynamics. Further, simulation elucidates a remarkable morphology-dependent hydrodynamic mechanism of boundary approach, hypothesised to be the cause of the well-established phenomenon of tip-first epithelial attachment of *Leishmania* promastigotes to the sandfly vector midgut.

## Highlights

• *L. mexicana* behaviour near a no-slip surface is studied via boundary element methods.
• Model flagellar beat for *Leishmania* promastigotes is identified from videomicroscopy.
• Idealised simulation of boundary approach shows dichotomy of deflection or collision.
• Repulsive surface forces do not induce stable boundary accumulation in this puller.
• Tip-first boundary approach may be promoted by morphology-dependent drag.

## Graphical abstract

**Figure:**
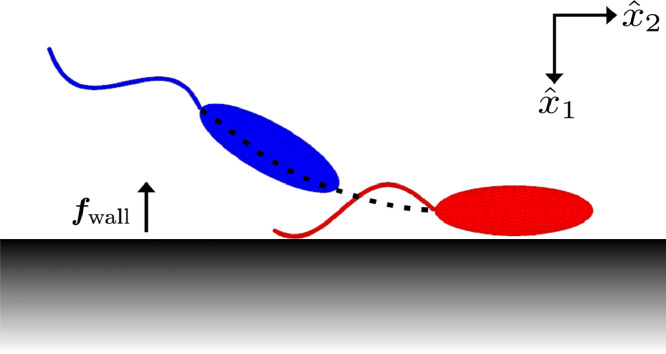

## 1 Introduction

**Fig. 1:**
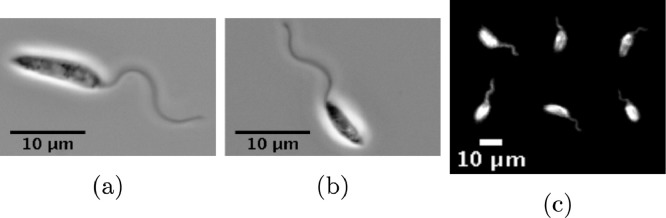
Sample frames from *L. mexicana* promastigote videomicroscopy, between two coverslips (a,b), and in the bulk, as presented in montage form for (c). We observe that the length scales of body and flagellum are approximately equal, with the cell body being approximately ellipsoidal in shape. Flagellar beating can be seen to be sinusoidal and planar in character, in both a confined environment and in the bulk.

The unicellular parasitic eukaryotes of the family Trypanosomatidae are the cause of many major human diseases including African trypanosomiasis and New World leishmaniasis (Herwaldt, 1999). Those of the genus *Leishmania*, transmitted to humans by the bite of a sandfly, affect around 4 million individuals globally (Herricks et al., 2017). A prominent cause of cutaneous leishmaniasis in the Americas, *L. mexicana* are a popular focus of recent research owing to their complete development cycle being observable *in vitro* (Bates, 1994). In the highly motile promastigote stage of their life cycle, a stage defined by morphology and as shown in Fig. 1, they utilise a single flagellum for locomotion, protruding from their anterior cell body and predominantly beating with a tip-to-base planar wave, the latter being common to all Trypanosomatidae (Branche, 2006, Gadelha, Wickstead, Gull, 2007, Goldstein, Holwill, Silvester, 1970, Holwill, McGregor, 1974, Holwill, McGregor, 1976, Johnston, Silvester, Holwill, 1979). Their viability in the sandfly vector midgut is thought to depend upon their ability to navigate effectively (Cuvillier et al., 2000), with it being widely accepted that their survival in the low-Reynolds number environment of the sandfly midgut is reliant upon attachment to the nearby epithelium (Bates, 2008, Dostálová, Volf, 2012). In fact, the precise driving mechanism of the tip-first boundary approach of *Leishmania* promastigotes remains unknown, and is hypothesised by Bates (2008) to simply be a naive consequence of their flagellum-first swimming direction, but the effects of potential hydrodynamic factors remain to be considered in detail. Contrastingly, in many *Leishmania*-sandfly pairings the mechanism of epithelial binding has been well-explored, evidenced to be dependent upon the major *Leishmania* surface glycoconjugate, *lipophosphoglycan* (LPG) (Butcher, Turco, Hilty, Pimenta, Panunzio, Sacks, 1996, Pimenta, Saraiva, Rowton, Modi, Garraway, Beverley, Turco, Sacks, 1994, Pimenta, Turco, McConville, Lawyer, Perkins, Sacks, 1992, Soares, Macedo, Ropert, Gontijo, Almeida, Gazzinelli, Pimenta, Turco, 2002, Soares, Margonari, Secundino, MacÊdo, Da Costa, Rangel, Pimenta, Turco, 2010). Following metacyclogenesis, and an accompanying change in LPG, the epithelial binding is reversed, resulting in the detachment of the promastigote from the midgut surface (Pimenta et al., 1992).

A direct consequence of locomotion via tip-to-base flagellar beating, *Leishmania* spp. are hydrodynamically classified as *pullers*, achieving propulsion by drawing fluid along the length of the flagellum before then pushing out the fluid at the sides. This is in contrast to *pushers*, such as human spermatozoa and *E. coli*, which perform the reverse action and are consequently propelled in the opposite direction (Lauga and Powers, 2009). Differences between the hydrodynamic properties of pushers and pullers have been well documented for the case of *squirmers*, swimmers of nearly constant shape with generated fluid flow at their boundary, a model classically applied to *Opalina* and other ciliated microorganisms (Blake, 1971b, Chisholm, Legendre, Lauga, Khair, 2016, Ishimoto, Gaffney, 2013). Further, within the classes of pusher and puller fundamentally different behaviours are observed, even for the simplest swimmers, as illustrated by the contrast between a force-dipole puller, which deflects from boundaries (Lauga and Powers, 2009), and the spherical puller squirmer, which swims stably near boundaries (Ishimoto and Gaffney, 2013). Hence refined models of cellular swimmers are required to elucidate their boundary dynamics, as illustrated by the rich boundary behaviours observed for flagellate pushers such as *E. coli* and mammalian spermatozoa, together with the biflagellate puller *Chlamydomonas*, in recent extensive work (Elgeti, Kaupp, Gompper, 2010, Fauci, McDonald, 1995, Ishimoto, Gaffney, 2015, Kantsler, Dunkel, Polin, Goldstein, 2013, Lauga, DiLuzio, Whitesides, Stone, 2006, Lopez, Lauga, 2014, Smith, Gaffney, Shum, Gadêlha, Kirkman-Brown, 2011, Smith, Gaffney, Blake, Kirkman-Brown, 2009a). However, corresponding studies of monoflagellated pullers, either observational or simulation-based, are comparatively lacking and hence there is extensive scope for the investigation of the boundary behaviours of a flagellated puller such as *L. mexicana*.

In particular, additional to their differing hydrodynamic classification, *Leishmania* promastigotes are also morphologically distinct from the better-studied pusher monoflagellates. Accumulation behaviours are reported to be sensitive to variations in swimmer morphology (Ishimoto, 2017), even for puller squirmers (Ishimoto and Gaffney, 2013), while appeal to time reversal symmetry to infer puller behaviour from pusher behaviour requires the same cellular morphology. Hence *Leishmania* swimming behaviour cannot be inferred from previous studies of swimmers, due to its distinct cell morphology, with the length scales of the flagellum and cell body approximately equal (at approximately ∼10µm, see Fig. 1). In contrast, for a typical human spermatozoon this ratio approaches one-tenth, with the spermatozoon cell body being substantially shorter than the attached flagellum (Katz, Overstreet, Samuels, Niswander, Bloom, Lewis, 1986, Wheeler, Gluenz, Gull, 2011).

The smaller cell body of such spermatozoa also enables the use of approximate analytic techniques such as resistive force theory in studying their motility (Johnson and Brokaw, 1979). This has been implemented classically for the spermatozoa of the sea urchin *Psammechinus* by Gray and Hancock (1955), where hydrodynamic interactions between the two cell components are either neglected or treated simplistically. Given the comparable scales of cell body and flagellum in *Leishmania*, such an approach is inappropriate (Johnson and Brokaw, 1979), with methods treating the flagellum and cell body comparably being more natural, and indeed, accurate. Thus, a full and high-accuracy numerical study is necessitated to fully capture the hydrodynamics and resulting behaviours of *Leishmania*.

The functional relevance of a beating flagellum to the promastigote is the control of spatial location. Hence we will examine the mechanics of *Leishmania* upon approach to, and movement away from, a boundary. Thus our aim is to consider how the cell may control its location in the sandfly midgut, in its need to both approach and leave the gut epithelium at different stages of its life cycle.

Hence, in this paper we will firstly detail digital capture for the flagellum waveform of *Leishmania mexicana* in a typical growth medium. We then seek a low-dimensional expansion of the observed kinematics via standard Fourier analysis (Ma, Klindt, Riedel-Kruse, Jülicher, Friedrich, 2014, Werner, Rink, Riedel-Kruse, Friedrich, 2014), and use a high-accuracy boundary element computational framework to perform a number of *in silico* experiments (Pozrikidis, 2002), our primary objective being to document the complex long-term behaviour of a virtual promastigote in the presence of a planar boundary. Further, we use beat-averaged phase planes to classify and quantify behaviours in the near and far-field of a boundary, drawing simulation-based behavioural comparisons with classical pushers such as the human spermatozoon. Thus our final objective is to examine the hypothesis that hydrodynamic interaction is sufficient for the tip-first boundary approach of *L. mexicana* promastigotes, and additionally that repulsive boundary interactions drive the separation of promastigotes from a boundary.

## 2 Methods

### 2.1 Videomicroscopy of *L. mexicana*

*Leishmania mexicana* high framerate videos were generated similarly to previously described in Wheeler (2017). Promastigote *L. mexicana* (WHO strain MNYC/BZ/62/M379) were grown in M199 supplemented with 10% FCS and 50 µM HEPES · HCl pH 7.4, and maintained in exponential growth between approximately 1 × 10^6^ and 1 × 10^7^ cells/ml. For high framerate videos, plain glass slides and coverslips were first blocked by immersion in 1% bovine serum albumen for 30 s, then washed with distilled water. An approximately 2 × 1cm^2^ rectangle was drawn on the blocked slide with a hydrophobic barrier pen, to which 1µl *L. mexicana* culture in logarithmic growth was added, then a blocked coverslip was added giving a ca.  ∼ 5µm sample depth. 200 and 400 frame/s videos between 4 and 9s long were captured with an Andor Neo v5.5 sCMOS camera using phase contrast illumination on a Zeiss Axio Observer inverted microscope with a 63 ×  N.A. 1.3 Plan-Neofluar objective (420881-9970-000) and a N.A. 0.55 long working distance condenser. Sample frames can be seen in Fig. 1.

For visualising *L. mexicana* waveforms in the bulk a 250 µm thick adhesive plastic square was applied to a glass slide to make a deep chamber, in which 10 µl *L. mexicana* culture was placed, then a coverslip added. Images were captured at a focal plane mid-way through the sample depth, using dark field illumination and a long working distance 10 ×  N.A. 0.45 Plan-Apochromat objective (1063-139). Many cells lay with their cell body and flagellum entirely in the focal plane, consistent with a planar flagellar beat (see Fig. 1c).

### 2.2 Determining flagellar kinematics

**Fig. 2:**
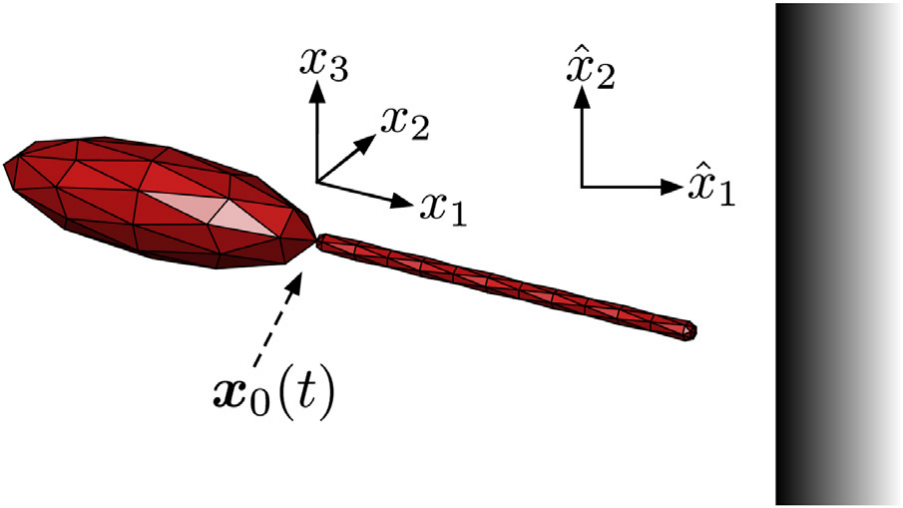
A 3-dimensional computational representation of the virtual promastigote with undeformed flagellum, where 80 triangular elements (red) have been used to mesh the cell body surface for illustration purposes (see Appendix B). The location of the flagellar attachment at time *t* is denoted ***x***_0_(*t*) in the laboratory frame, and is the origin of the cell-fixed reference frame whose axes, *x*_1_*x*_2_*x*_3_, are depicted. A solid boundary lies in the laboratory frame plane ${\hat{x}}_{1}=$ constant. (For interpretation of the references to colour in this figure legend, the reader is referred to the web version of this article.)

Flagellar kinematics were extracted via automated analysis in the ImageJ macro language, relative to a cell-fixed reference frame with coordinates $x=(x_{1},x_{2},x_{3})$. This frame is defined as having *x*_1_ directed along the axis joining the body centroid to the visible base of the flagellum, with the base being placed at the origin and having coordinates ***x***_0_ in the inertial laboratory frame (see Fig. 2). In a similar analysis of mammalian spermatozoa (Ishimoto, Gaffney, 2014, Smith, Gaffney, Gadêlha, Kapur, Kirkman-Brown, 2009b), a tangential attachment of the flagellum to the cell body was assumed due to the presence of structural components, such as outer dense fibres, which provided sufficient information to rotate the captured data into the cell-fixed frame. Indeed, this would be appropriate for *Leishmania* at the true attachment site inside the flagellar pocket, where microtubule structures bind the flagellum to the cell body (Lacomble, Vaughan, Gadelha, Morphew, Shaw, McIntosh, Gull, 2009, Lacomble, Vaughan, Gadelha, Morphew, Shaw, McIntosh, Gull, 2010). However, our captured data provides an exterior view at resolution such that the perceived flagellar attachment appears free, and at a site that we will refer to as the base, distinct from the true flagellar attachment zone in the flagellar pocket. Therefore, relaxing the constraint of tangential attachment is suitable here, and indeed this provides good agreement with observed waveforms. Hence, the cell body rotation is instead used to determine the cell-fixed frame orientation in the inertial frame, with the flagellar base ***x***_0_ being the centre of rotation here and throughout. Approximate wavelengths and amplitudes were computed similarly to Gadelha et al. (2007), where appropriate, along with an approximation to the cell body length. A standard decomposition of the resulting waveform data into Fourier modes was then performed (Ma, Klindt, Riedel-Kruse, Jülicher, Friedrich, 2014, Werner, Rink, Riedel-Kruse, Friedrich, 2014), where an expansion of low dimension was sought for use in numerical simulations.

### 2.3 Governing equations

The small scale dynamics of *L. mexicana* promastigotes in a Newtonian fluid of viscosity *μ* is governed by the incompressible Stokes equations (see Appendix A), with a Reynolds number on the order of $10^{-3},$ using typical length and velocity scales given in Wheeler et al. (2011), Wheeler (2017). For a given surface *S*, which typically will represent the promastigote surface, we have the following non-dimensional integral representation for the instantaneous flow velocity ***u*** relative to the inertial frame at a point ***x***^⋆^ on the surface, with coordinates given in the body-fixed frame and following Pozrikidis (2002),

(1) $$\begin{array}{ccc} u_{j}\left(x^{★}\right) & = & -\frac{1}{4\pi \mu }\int_{S}G_{ij}\left(x,x^{★}\right)f_{i}\left(x\right)\;dS\left(x\right)\\ & & +\;\frac{1}{4\pi }\int_{S}^{PV}u_{i}\left(x\right)T_{ijk}\left(x,x^{★}\right)n_{k}\left(x\right)\;dS\left(x\right). \end{array}$$

Here, *G_ij_* and *T_ijk_* are velocity and stress Green’s functions of 3-dimensional Stokes flow, ***n*** is the surface normal directed into the fluid, ***f*** denotes the surface traction, and ∫^*PV*^ denotes a principal value integral in the second summand. The velocity may be decomposed into background, cell and disturbance velocities as in Ishimoto and Gaffney (2014), where the cell linear and angular velocities, ***U*** and **Ω**, are a priori unknown in the inertial frame and must be solved along with the boundary tractions, subject to additional force and torque-free constraints.

### 2.4 The virtual promastigote

In order to model the swimming behaviour of *Leishmania* we introduce a neutrally-buoyant *virtual promastigote*, which here will have an idealised geometry that is similar to wild-type promastigotes (see Figs. 1 and 2). In particular, we construct our idealised promastigote using an axisymmetric prolate ellipsoid to represent the cell body, which differs slightly from observed *L. mexicana* promastigotes, as the latter typically exhibit limited body curvature along their long axis (see Fig. 1 for typical examples). With reference to the non-dimensionalisation scales used in Appendix A, we prescribe a non-dimensional body length of 1.1, with circular cross sections of diameter 0.35, consistent with a typical promastigote length scale of 10 µm and corresponding to a non-dimensional flagellum length of 1.3. In turn, we model the latter by introducing a slender capped cylinder of non-dimensional width 0.03 that attaches to the body at the origin of the cell-fixed frame. The flagellum shape in the cell-fixed frame is described by a general parameterisation

(2) $$\begin{array}{c} x=H\left(\xi ,t\right)\;,\;\xi \in [0,\xi^{★}\left(t\right)]\;, \end{array}$$

where the quantity *ξ*^⋆^(*t*) is chosen such that the arclength of the flagellum is conserved, and additionally we enforce that H(0,t)=0, ensuring flagellar attachment occurs at the same location on the body for all times *t*. Where it is appropriate to define a beat plane in the cell-fixed frame, we will assume without loss of generality that such a plane is spanned by unit vectors in the *x*_1_, *x*_2_-directions, so that there is no beating in the *x*_3_ coordinate direction (see Fig. 2). Flagellum material velocities in the inertial frame at a given time *t* are approximated from positional information using a central differences scheme optimised for double precision arithmetic (Nocedal and Wright, 1999).

### 2.5 Numerical scheme

Given the instantaneous velocity of the flagellum in the cell-fixed frame, we proceed to solve the boundary integral equations of 3-dimensional Stokes flow over the discretised virtual promastigote surface, closing the system with the conditions of force and torque–free swimming, which are appropriate in the inertialess limit of Stokes flow. Geometry, surface tractions and surface velocities are interpolated using a mesh of *n* nodes, yielding a linear system of 3n+6 equations in 3n+6 unknowns, including the components of swimming velocity ***U*** and angular velocity **Ω** (for mesh details see Appendix B). We additionally enforce, without loss of generality, that the normal boundary traction has a surface mean of zero, eliminating the pressure non-uniqueness inherent in Stokes flow, and solve the resulting system for the virtual promastigote velocities and surface tractions. Throughout, we use the Blakelet for the integral kernel *G_ij_* in Eq. (1) (Blake, 1971a), along with the accompanying form of *T_ijk_*, which ensures that the solutions satisfy a no-slip condition on a specified planar boundary. Denoting coordinates in the laboratory frame by $\hat{x}=\left({\hat{x}}_{1},{\hat{x}}_{2},{\hat{x}}_{3}\right),$ we typically specify this stationary boundary as ${\hat{x}}_{1}=0$.

Having computed instantaneous promastigote velocities at a time *t*, we use Heun’s method with timestep *dt* to update the position and orientation of the cell in the inertial frame, as detailed in Smith et al. (2009a), with positional evolution of the flagellum base point ***x***_0_ following the predictor-corrector scheme

(3) $$x_{0}\left(t+dt\right)=x_{0}\left(t\right)+\frac{dt}{2}\left[U(t)+U(t+dt)\right]$$

and cell orientation being dealt with similarly. An adaptive timestepping scheme is employed in order to increase accuracy when approaching the boundary, where velocities are expected to be highly sensitive to boundary separation and body configuration.

Noting that surface interactions such as steric forces often occur between cells and substrates, but also are highly variable between different solutes and substrates (Klein et al., 2003), we proceed to additionally consider a surface force. While an attractive surface force will simply tend to induce binding once a cell is sufficiently close to a boundary wall, the impact of a repulsive potential is ambiguous a priori, with the potential to reflect the cell away from the boundary or to induce stable swimming for a cell that would otherwise crash into the boundary. Hence we consider a repulsive boundary force via a surface potential, as introduced by Ishimoto and Gaffney (2016) and motivated by Klein et. al’s measurements (Klein et al., 2003). The resulting force in non-dimensional form is given by

(4) $$f_{\text{wall}}\left(x\right)=g\frac{e^{-d/l}}{1-e^{-d/l}}n\;,$$

where *d* is the boundary separation, ***n*** is the outward-facing normal of the boundary, and $l=0.02,\;g\propto \hat{\mu }/\hat{T}$ are the effective range and strength of the force, chosen such that a strong short-range repulsion is represented, with strength scaling with dimensionless viscosity $\hat{\mu }$ and beat period $\hat{T}$.

Our implementation was verified in free-space against Ishimoto and Gaffney (2014) and by reproducing the Jeffery’s orbits of ellipsoidal particles (Jeffery, 1922), whilst the implementation of the Blakelet was compared with the software library BEMLIB (Pozrikidis, 2002).

### 2.6 Construction of phase planes

**Fig. 3:**
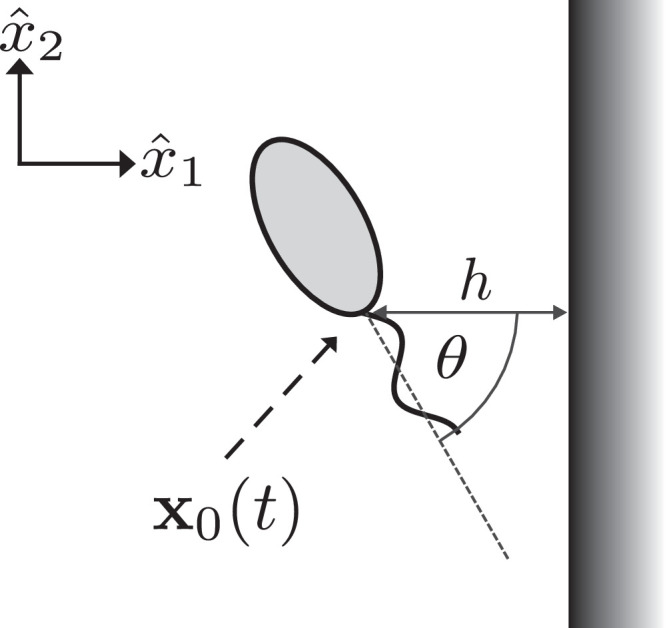
Schematic showing the planar configuration of a virtual promastigote, where boundary separation is denoted *h*, being measured from the flagellum attachment point ***x***_0_. The clockwise angular displacement of the cell-fixed frame from the laboratory frame is denoted *θ*, as shown, such that θ=0 corresponds to perpendicular approach towards the boundary.

In an effort to gain a more complete picture of the virtual promastigote dynamics without performing numerous costly individual simulations, we attempt to simplify the dynamics via its restriction to a plane autonomous system, as shown parameterised in Fig. 3. Specifically, we proceed by equating the third coordinate vectors of the inertial and cell-fixed frames, so that motion and beat plane are confined to the plane ${\hat{x}}_{3}=0,$ without loss of generality. We additionally average over a single beat period, as in Ramia et al. (1993), enabling a parameterisation by boundary separation and orientation alone, which we denote by *h* and *θ* respectively (see Fig. 3). We define the separation to be the distance from the flagellar attachment point to the wall, and the orientation to be the clockwise angle between the body-fixed *x*_1_-axis and the boundary normal. We can then form the representation

(5) $$\begin{array}{ccc} \dot{h} & = & F(h,\theta ),\\ \dot{\theta } & = & G(h,\theta ), \end{array}$$

where *F* and *G* represent the process of boundary element simulation and subsequent phase averaging. Note that we may identify $-\dot{h}\equiv {\bar{U}}_{1}$ and $-\dot{\theta }\equiv {\bar{\Omega }}_{3},$ the phase-averaged linear velocity in the ${\hat{x}}_{1}$-direction and the rate of rotation about the ${\hat{x}}_{3}$-axis respectively.

## 3 Results

### 3.1 *L. mexicana* exhibit simple flagellar kinematics

**Fig. 4:**
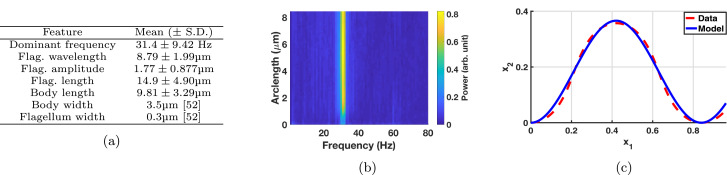
(a) Typical cell parameters as computed from an analysis of *L. mexicana* videomicroscopy of N=126 cells, along with body and flagellum widths from Wheeler et al. (2011). The flagellar beat amplitude is noted to be approximately half the typical cell body width. (b) The Fourier power spectrum for a sample cell. A strong dominant frequency band can be seen around 28Hz, present for the entire length of the flagellum. Power is shown here in arbitrary units, and arclength is measured from the flagellum base. (c) A flagellar waveform extracted from video microscopy (red, dashed) plotted against a model beat with a single sinusoid (blue, solid), showing excellent agreement both in space and in time (time not shown). (For interpretation of the references to colour in this figure legend, the reader is referred to the web version of this article.)

Analysis of the temporal Fourier spectra of the *L. mexicana* flagellar beat for a sample of N=126 cells between two cover slips revealed a single prominent planar beat frequency in the range of 26–34 Hz, clearly observable along the entire flagellum length (see Fig. 4b). The lack of other significant modes suggests a decomposition into a single sinusoid is appropriate for representing the flagellar beat. Thus we opt to define the non-dimensional beat parameters of amplitude, wavelength and frequency, denoted *A, λ* and *f* respectively, and assume the functional form

(6) $$\begin{array}{ccc} x_{1}\left(\xi ,t\right) & = & \xi ,\\ x_{2}\left(\xi ,t\right) & = & A\left[\sin\left(\frac{2\pi }{\lambda }\xi +2\pi ft\right)-\sin\left(2\pi ft\right)\right],\\ x_{3}\left(\xi ,t\right) & = & 0 \end{array}$$

in the cell-fixed frame. Under this assumption, amplitudes and wavelengths were approximated, with the averaged results being shown in Fig. 4a. Here we set A=0.18,λ=1.3 and f=2.8 for use in simulations, recovering a typical long-wavelength flagellar beat (see Supplementary Movie 1) and noting that the results that follow are not sensitive to variations in these parameter choices. Good agreement between the model flagellar beat and that extracted from data is shown in Fig. 4c, demonstrating a remarkably simple flagellar kinematics, not dissimilar to that of *L. major* and the classically-studied *Crithidia oncopelti*(Gadelha, Wickstead, Gull, 2007, Holwill, McGregor, 1974). Additionally, it is noted that whilst this planar beat pattern was seen in confined promastigotes (see Section 2.1), such beating is also observed in the bulk and no non-planar beating is exhibited (see Fig. 1c). Furthermore, from analysing observations of the human spermatozoon, it has also been reported that monoflagellate beating is unchanged near to a boundary to the resolution that can be observed with typical microscopy (Ishimoto and Gaffney, 2014, Appendix A). Therefore we adopt our model beat pattern both in the far and near-field of boundaries, and assume that there is not significant variation in this waveform due to hydrodynamic boundary effects.

### 3.2 Virtual promastigote beat plane aligns towards the perpendicular

**Fig. 5:**
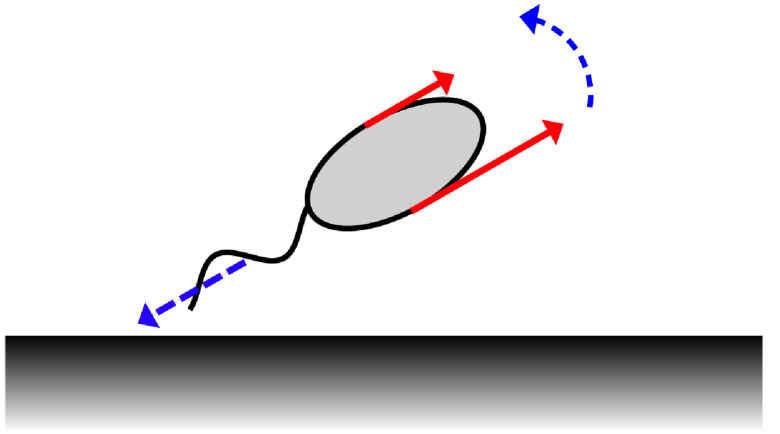
Physical mechanism of morphology-dependent drag-based rotation. The no-slip boundary induces increased drag on the nearside portion of the cell in comparison to the far side (red, solid), generating a net torque which causes cell reorientation, in addition to the typical promastigote movement towards the boundary (blue, dashed). (For interpretation of the references to colour in this figure legend, the reader is referred to the web version of this article.)

Long-time simulations of virtual promastigotes revealed a tendency to align their beat plane normal to the boundary when *h*, the cell-boundary distance, is sufficiently small. This may be explained by a simple torque balance argument, especially due to the cell-body size, and exemplifies a general observation that pullers tend to align perpendicular to boundaries (Lauga, Powers, 2009, Spagnolie, Lauga, 2012). In this instance, and with reference to Fig. 5, the no-slip boundary induces increased drag on the near-side of the cell body in comparison to the far side. This difference results in a torque, which when combined with the constraint of torque-free swimming drives reorientation towards the perpendicular, significantly more so than is present when the body size is reduced. The difference in hydrodynamic drag across the virtual promastigote may be explicitly computed by prescribing an instantaneous cell velocity and orientation in place of the force-free and torque-free conditions, the results of which are shown in Table 1 for various swimmer configurations and body length scales. These figures demonstrate the existence of a notable drag difference between near and far sides of the swimmer, and in particular the dependence of this difference on body size, supporting the conclusion that increased cell body size is a factor in the hydrodynamic promotion of promastigote reorientation. This behaviour, and likewise the observations that follow, are observed to be robust to small changes in cell aspect ratio and beat parameters (see Appendix C).

**Table 1:** Percentage increase in hydrodynamic drag between the bulk and wall-facing sides of a virtual promastigote moving along the axis of the flagellum. Relative drag difference is shown for various configurations in phase space in the first section of the table for a virtual promastigote with typical morphology as described in Section 2.4, with the drag differential being greatest for low separations *h*. The dependence of this drag difference on swimmer body morphology is captured by the second part of the table, where we fix θ/π=0.4 and vary the length of the swimmer body whilst preserving its aspect ratio. Increased body length scale is seen to significantly increase the relative drag difference, and thus a strong dependence of the resultant forcing of the swimmer on body morphology is highlighted. Boundary separation *h* and body length are non-dimensional, and configurations that result in intersection with the boundary are omitted.

		*θ*/*π*				Body length			
		0.2	0.3	0.4	0.5	0.11	0.44	0.77	1.1
*h*	0.6	·	·	11.0	13.5	2.8	3.9	6.9	11.0
	0.8	·	8.7	7.2	8.3	1.4	2.2	4.3	7.2
	1.0	·	5.1	5.1	5.7	0.9	1.5	3.0	5.1
	1.2	3.5	3.9	3.9	4.1	0.6	1.1	2.3	3.9

### 3.3 Virtual promastigotes reorient to promote boundary collision via distal flagellar tip

**Fig. 6:**
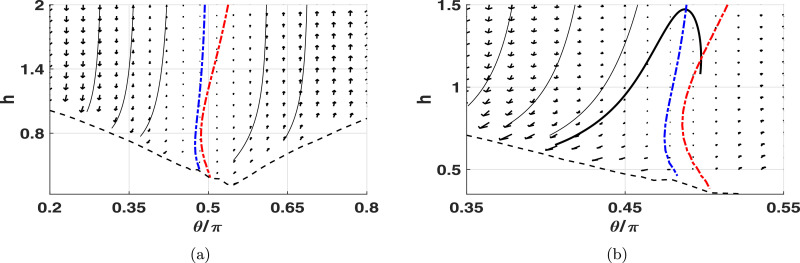
Beat-averaged phase planes approximating virtual promastigote motion near a boundary. Sample trajectories are shown as black lines, with the black dashed line separating off the region where configurations intersect with the boundary. Nullclines of the separation *h* and orientation *θ* are shown as dashed lines (blue and red respectively), with the *h* nullcline approaching θ=π/2 as *h* becomes large. (a) We observe a full spectrum of dynamics, with boundary collision occurring in the left half of the phase plane, and reorientation away from the boundary in the right half. For cells approaching from the far-field (*h* → ∞, |*θ*| < *π*/2), the trapping regions of the phase plane show that boundary collision occurs for most values of *θ*, with deflection appearing possible only for *θ* ≈ *π*/2. The latter observation is confirmed by long-time simulation, where deflection may be observed if θ=π/2 initially. The phase plane has no fixed points or periodic orbits, corresponding to a lack of stable boundary swimming. (b) Higher resolution phase plane highlighting drag-based reorientation. Trajectories can be seen to curve rapidly in the direction of decreasing *θ* as they approach the boundary. A sample computed trajectory (heavy line) appears to exhibit a non-monotonic change in *h*. (For interpretation of the references to colour in this figure legend, the reader is referred to the web version of this article.)

Upon a collision-bound approach to a planar boundary, in the absence of additional repulsive surface forces, we observe the remarkable reorientation of the virtual promastigote such that the distal tip of the flagellum is promoted as the point of contact. This is visible in numerous individual simulations, the behaviour also being captured by the phase plane (Fig. 6b), where we see a rapid change in angle *θ* when in close proximity to the boundary. This may again be partially explained by the general tendency of pullers to align perpendicular to a wall, but the large magnitude of the effect suggests that it is also resultant of the same drag-based mechanism as the beat plane reorientation above. Indeed, this is confirmed by repeating simulations with greatly-reduced body size, where the effects are seen to be substantially decreased, consistent with the quantitative observations shown in Table 1. Thus both beat plane orientation and the specifics of boundary approach are dependent upon virtual promastigote morphology, and together result in the flagellar tip being the primary point of surface contact.

### 3.4 Swimming is unstable in the absence of a surface force

As described in Section 2.6, we compute a phase plane in order to deduce long-time behaviour (see Fig. 6a). Partitioning phase plane trajectories by the *h* nullcline (which approaches θ=π/2 in the far-field, depicted blue, dashed), we observe that almost all virtual promastigotes approaching the boundary from the far-field will eventually collide with the boundary (see Supplementary Movie 2). Similarly, almost all trajectories that initially face away from the boundary exhibit a monotonic increase in boundary separation and relative angle, with those that are sufficiently close to the boundary undergoing deflection, where *θ* initially increases and then approaches a constant value as promastigote-wall separation grows.

However, the phase plane shows a region where non-monotonic change in *θ* appears plausible (see Fig. 6b), suggesting that those promastigotes beginning on trajectories in the region between the *h* and *θ*-nullclines will initially move away from the boundary, but will subsequently undergo reorientation towards the surface and eventually collide with it. Contrastingly, such behaviours are not observed in full simulations, however this is not a significant conflict given the magnitude of the error introduced by the phase plane averaging process. This error is typically of the order $10^{-6},$ comparable to the $\dot{\theta }$ values seen in this limited region of the phase plane near the nullclines, therefore the approximate behaviour need not accurately reflect the full dynamics local to this region. Thus, overall we see a dichotomy of behaviours exhibited by the virtual promastigote, those of collision with or deflection away from the boundary, with no mechanism for stable boundary swimming. This is confirmed by long-time simulation of virtual promastigotes with *θ* ≈ *π*/2 initially, which orient away from the boundary rather than swimming stably (see Supplementary Movie 3).

**Fig. 7:**
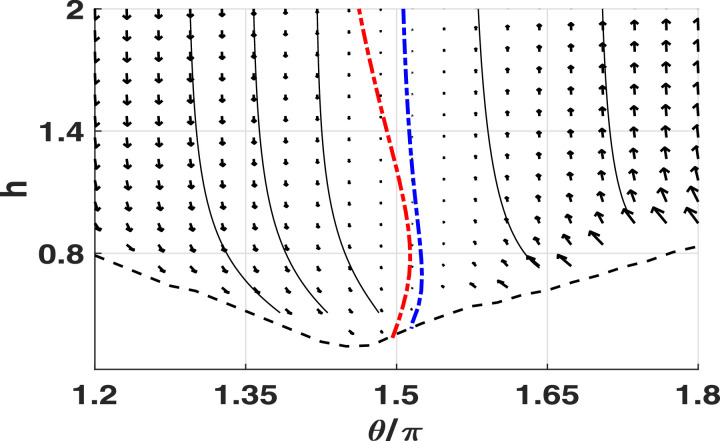
Beat-averaged phase plane approximating the motion near a boundary for a virtual pusher with the same morphology as the virtual promastigote of Fig. 2. Sample trajectories are shown as black lines, with the black dashed line separating off the region where configurations intersect with the boundary. Nullclines of the separation *h* and orientation *θ* are shown as dashed lines (blue and red respectively), with the *h* nullcline approaching θ=3π/2 as *h* becomes large. Having reversed the propagation direction of the flagellar beat, we recognise the time-reversed behaviour of the virtual promastigote, subject to a shift in *θ* (cf. Fig. 6a). Noting the sign of $\dot{\theta }$ near the *θ*-nullcline, we see the stable attractor of *θ* ≈ 3*π*/2. (For interpretation of the references to colour in this figure legend, the reader is referred to the web version of this article.)

In order to compare these behaviours against an appropriate pusher, we reverse the direction of beat propagation in the flagellum and simulate the resulting motion. The behaviour of this pusher is captured by a phase plane (see Fig. 7), where we observe that *θ* ≈ 3*π*/2 is a stable attractor of the system, corresponding to swimming parallel to the boundary. In long-time simulations of the full system we in fact observe stable boundary swimming at a constant separation *h*, which differs slightly from the beat-averaged system due to the previously-described beat cycle averaging errors near the system nullclines. In explanation of this stable swimming with reference to the virtual promastigote, we firstly note that a change in beat propagation direction is in fact equivalent to a reversal of time in the flagellar kinematics of Eq. (6), subject to a phase shift. As time appears only as a parameter in the governing equations, the solution to the time-reversed problem is simply the reversal of the original problem. Therefore we recover the reversed behaviour of the virtual promastigote in the behaviours of this pusher, and hence observe stable parallel swimming in the place of unstable motion. Additionally, we examine the effects of significant decrease in cell body size by further modifying the virtual promastigote, reducing the body volume by two orders of magnitude, and repeating the above analysis. From this we note that the stable swimming of the altered pusher may still be observed, albeit at a different boundary separation *h*, whilst the puller behaviour remains unstable. Thus, our results suggest that hydrodynamic classification is a more significant factor than morphology in determining the stability of boundary swimming.

### 3.5 Repulsive surface forces do not give rise to stable boundary swimming

**Fig. 8:**
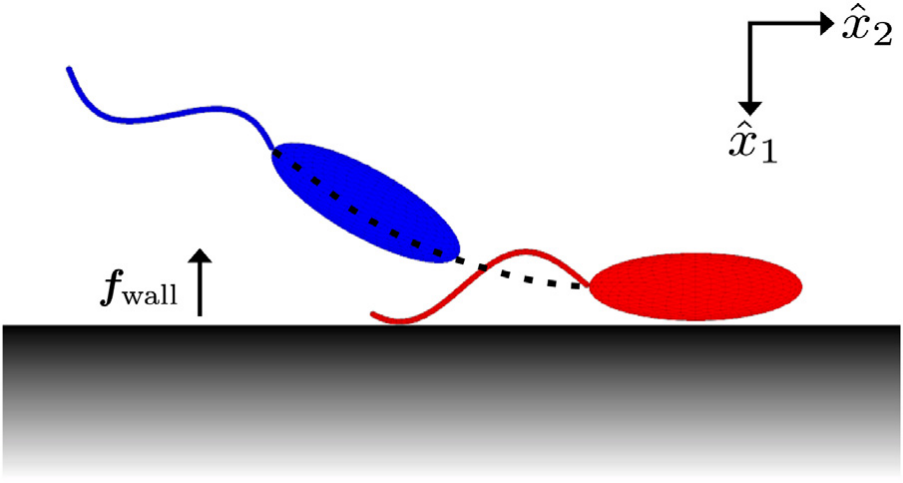
The simulated motion of a virtual promastigote in proximity to a boundary, accounting for a repulsive surface potential. The computational mesh is shown for the initial configuration (red), parallel to the boundary, and the configuration after ca. 1.4s (blue). The approximate time-averaged path of the attachment point is also shown (black, dashed). We observe that the promastigote orients away from the boundary, and thereafter moves off into the bulk. See Supplementary Movie 4 for full motion. (For interpretation of the references to colour in this figure legend, the reader is referred to the web version of this article.)

The repulsive surface potential of Section 2.5 is introduced to the system. Due to the short range over which the resulting repulsive force is non-negligible, the previous phase plane analysis that does not account for the surface force holds throughout most of the phase space. However, due to the varying distance of the flagellum from the boundary throughout a single beat period, phase averaging is not appropriate to determine the near-boundary dynamics. Hence numerous long-time simulations were performed (see Fig. 8 for an example) to ascertain the motion of the virtual promastigote when in close proximity to the boundary, following which the phase plane of Fig. 6a is used to examine further behaviour.

One might envisage the existence of a periodic motion, with the torque induced by the surface potential orienting the cell into a configuration where it will again collide with the boundary, and such motion repeats ad infinitum. This phenomenon could not be observed for all conducted *in silico* experiments with typical parameter values and cell configurations, which we reason is due to the strong repulsive boundary character.

We observe that the only exhibited behaviour is deflection away from the boundary, with the surface repulsive force causing reorientation of the promastigote such that it falls into the deflective regime, which is observed to be unchanged for reasonable variation in force strength due to the short-range nature of the surface potential. Thus the inclusion of a repulsive surface potential of physically-reasonable character to model boundary surface forces does not give rise to stable boundary swimming of virtual promastigotes, and instead promotes their eventual deflection.

Reversing the direction of beat propagation we examine the near-boundary behaviour of the morphologically-equivalent pusher, which due to the repulsive surface force may not be inferred by time reversal of the puller behaviour. In contrast to the deflection observed for the puller, in this case we in fact see that the swimmer aligns approximately parallel to the boundary, approaching a stable separation from the wall after an initial period of transition, qualitatively similar to the non-deflective behaviour of small-bodied pushers and as exemplified in Supplementary Movie 5. Thus our results further evidence the significance of hydrodynamic classification in determining boundary behaviours, and suggest in this case the subdominance of body morphology in effecting stable boundary swimming.

## 4 Discussion and conclusions

In this work we have identified and described a novel model flagellar waveform of *L. mexicana* promastigotes, observing a simple planar dynamics in N=126 cells that lends itself to simple parameterisation and subsequent simulation. Using this model beat pattern we have observed and explored the boundary behaviours of a virtual axisymmetric promastigote, an idealised hydrodynamic puller with a significant body lengthscale.

We have seen that swimming near a boundary in the absence of surface forces is unstable for virtual promastigotes, with trajectories resulting either in immediate deflection away from the boundary or eventual collision with the surface. This behaviour may not be deduced from previous observations of puller microswimmers, owing to the swimmer’s distinct cell morphology and the reported sensitivity of boundary behaviours to swimmer geometry (Ishimoto, 2017). Here, promastigotes that initially swim away from a boundary may be captured if sufficiently close, undergoing reorientation which results in their collision with the surface. Drawing comparison with *in vivo* promastigotes, this may provide hydrodynamic explanation for the epithelial attachment observed in the sandfly midgut that precedes promastigote metacyclogenesis, established to be necessary for the survival of *Leishmania* promastigotes (Bates, 2008, Dostálová, Volf, 2012).

Further, we have noted that time-reversibility of the governing equations allows us to immediately compare the dynamics of pushers and pullers near boundaries in scenarios without repulsive surface forces. Whilst our results have shown that no stable boundary swimming occurs in the case of our virtual promastigote puller, we observed the stable motion of morphologically-equivalent pushers, with the latter swimming parallel to the boundary. This is in agreement with the known behaviour of the smaller-bodied human spermatozoa, a pusher that can maintain stable parallel swimming next to a planar boundary (Elgeti, Kaupp, Gompper, 2010, Fauci, McDonald, 1995, Smith, Blake, 2009, Smith, Gaffney, Blake, Kirkman-Brown, 2009a). A significant reduction in cell body size is not observed to drastically alter the behaviour of the virtual pusher, with only the stable swimming height being affected. Thus our results show that the stable boundary swimming of monoflagellates in quiescent fluid is highly dependent on the method of locomotion, and less so to specific changes in morphological scales.

We have also seen that a change in surface character, from a scenario in the absence of surface forces to one with a short range surface force, reduces the prevalence of surface-bound trajectories, with those that would previously have ended in collision now being reoriented into the deflective regime. This promotion of deflective behaviour in the presence of additional repulsive effects is consistent with wild-type promastigotes, where a change in LPG during metacyclogenesis is thought to result in cell detachment from midgut epithelium, followed by taxis towards the sandfly foregut (Pimenta et al., 1992). Indeed, we conjecture that this taxis is aided by the transfer of promastigotes into the bulk and away from the no-slip boundary, so that cells are more susceptible to convection by background flows. Such flows may be associated with sandfly regurgitation, stimulated by the parasite’s production of promastigote secretory gel in their infective form (Bates, 2007). Thus the hydrodynamic interaction behind the deflection of virtual promastigotes may be responsible for *in vivo* movement of promastigotes into the bulk, and could therefore facilitate the transmission of the infective form of the parasite from vector to host.

However, whilst we have noted that stable boundary swimming of virtual promastigotes does not occur with a repulsive surface potential, this is not the case for the human spermatozoon (Ishimoto, Gaffney, 2014, Smith, Gaffney, Gadêlha, Kapur, Kirkman-Brown, 2009b). Differing in both hydrodynamic classification and cell morphology, it is not clear in the context of the presented results which feature drives the contrasting behaviours observed when accounting for short range surface forces, as time-reversibility is lost due to the boundary force. However, the above results indicate that cell morphology may not have significant impact in general on stable surface swimming, thus the observed behavioural differences between virtual promastigotes and human spermatozoa are hypothesised to be primarily resultant of the contrasting methods of locomotion.

Remarkably, we have observed a morphology-dependent mechanism for the promotion of tip-first boundary collision for virtual promastigotes near non-repulsive boundaries. We hypothesise that this mechanism may provide an explanation for the observed behaviour of *in vivo* promastigotes, where flagellum-first attachment is well-documented. This behaviour lacks an evidenced driving mechanism, though it has been postulated to be due to the flagellum-first nature of *Leishmania* swimming (Bates, 2008). Here *in silico*, our study of virtual promastigotes suggests a refined mechanism, whereby the comparatively large body size of the promastigote results in the emergence of drag-based reorientation of notable magnitude, highly dependent on body length scale, aligning the virtual flagellum such that the distal tip initiates boundary contact.

In Appendix C we have examined the behavioural effects of changing body length scale and aspect ratio, demonstrating that reported behaviours are robust to typical observed variation in these morphological parameters. There remains significant scope in future work to relax the assumption of body axisymmetry in order to more accurately model *Leishmania* promastigotes, and thus determine the impact of symmetry-breaking body geometry on boundary behaviours. One might expect there to be a significant dependence of behaviour on aspects of cell morphology, with certain realistic *Leishmania* promastigote body curvatures breaking all symmetry and thus adding further complexity to the dynamics. Additionally, the classification of boundary and general swimming behaviours for the different morphologies of promastigotes observed in sandflies, which is more diverse than in culture, is likely to be highly relevant in the continued study of *Leishmania* spp. and in the general investigation into hydrodynamic pullers with flagellum-scale cell bodies.

In summary, we have investigated in detail the boundary behaviours of a flagellated puller, a virtual *L. mexicana* promastigote equipped with a determined planar tip-to-base beat pattern, and have observed that stable boundary accumulation does not feature amongst the range of exhibited behaviours, irrespective of the inclusion of repulsive surface forces. Instead, long-time promastigote behaviour may be divided into two distinct categories based on initial location and orientation: those that are deflected away from the boundary, and those that collide tip-first with the boundary. However, whether or not these behaviours are truly representative of *Leishmania* promastigotes in their microenvironments requires further exploration. Nevertheless, our results suggest that the observed behaviour in the sandfly vector midgut may be explained by the hydrodynamic interactions between a promastigote and a boundary, enabling cell attachment and subsequent detachment at life cycle stages appropriate for *Leishmania* survival and virulence. In particular, we have seen that boundary collision via the distal tip of the flagellum is promoted mechanically in virtual promastigotes by a combination of a large cell body and tip-to-base beating, evidencing a remarkable morphology-dependent hydrodynamical mechanism of boundary approach.

## Figures and Tables

**Fig. C1:**
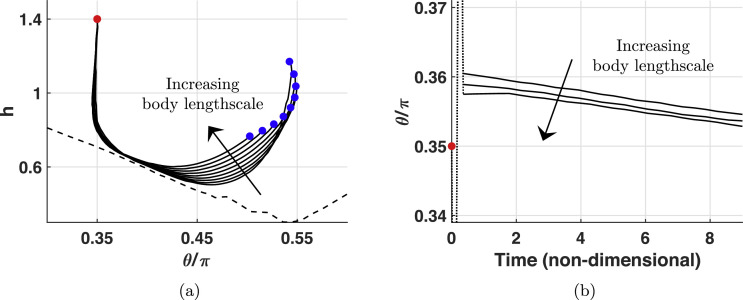
Simulated swimming of virtual promastigotes with differing body length scales. (a) Beginning from a sample initial condition of (θ,h)=(0.35π,1.4), with the initial location in phase space being shown in red and the simulation endpoints in blue, the smoothed paths of virtual swimmers are depicted as black curves. We simulate virtual promastigotes with a modified body length scale, where the aspect ratio of the body is kept fixed and the length of the body is sampled from a range of 7–17µm, representing typical variation in *Leishmania*. The black dashed line approximately separates off configurations that represent intersection with the boundary. We observe qualitatively-unchanged swimming behaviour in the presence of a repulsive surface potential, with reduced deflection being seen for larger-bodied swimmers. (b) Simulating the swimmers in the absence of short range boundary forces, halting prior to boundary contact, shown is a time series plot of the evolution of *θ*/*π*, with the initial configuration of all cells being (θ,h)=(0.35π,1.4) and length scales sampled from the same range as in (a). Greater alignment towards the boundary is observed for swimmers with increased body length scales. The dotted section of the curve shows an initial unsmoothed transient, with the common initial value highlighted in red. (For interpretation of the references to colour in this figure legend, the reader is referred to the web version of this article.)

## Appendix A Incompressible Stokes equations

Briefly stated, the dimensional Stokes equations for an incompressible Newtonian fluid with velocity field ***u*** and pressure *p* are given by

(A.1) $$\mu \nabla^{2}u=\nabla p\;,\;\nabla \cdot u=0\;,$$

where *μ* is the dynamic viscosity of the fluid. We non-dimensionalise with lengthscale L=10µm and velocity scale U=1µm s${}^{-1},$ and scale pressure with *μU*/*L*, taking the advective timescale *L*/*U* and using the dynamic viscosity of water at 25 °C. For typical *L. mexicana* values this gives a Reynolds number on the order of $10^{-3},$ so the inertia less limit of Stokes equations is appropriate here. Treatment of the non-dimensionalised equations follows Pozrikidis (2002), using a standard Green’s function approach to form the 3-dimensional boundary integral equation over a 2-dimensional surface, as given in Eq. (1). This equation is then applied to the surface of the virtual promastigote and discretised, with the resulting linear system being solved for cell velocities ***U*, Ω** given the prescribed flagellar kinematics, together with the force and torque conditions on the swimmer.

## Appendix B Meshing the virtual promastigote

Both icosahedral and triangulated-cubic meshes were used for the discretisation of the virtual promastigote body. Subdivision was performed by the bisection of existing edges, increasing the element count by a factor of 4 per subdivision. For the flagellum, a cylindrical mesh with spherically-capped ends was used, with the vertices being constructed in equispaced circular bands and triangular elements constructed between them. Typically in simulations a mesh of 320 elements was used for the cell body, and 324 for the flagellum. High sensitivity of simulation results on mesh geometry was noted, with a low resolution symmetry-breaking icosahedral mesh producing artefacts in long-time simulations. Thus simulation results were verified at higher mesh resolutions, where artefacts were of negligible magnitude.

## Appendix C Effects of body morphology

Given the reported variation in *Leishmania* promastigotes (Ambit, Woods, Cull, Coombs, Mottram, 2011, Wheeler, Gluenz, Gull, 2011), we examine the effects on swimming of altering the morphological parameters describing the swimmer body. With the body length of typical promastigotes varying approximately between 7 and 17µm (Wheeler et al., 2011), we simulate the motion of modified virtual promastigotes with body lengths sampled from this range. Here we fix the body aspect ratio to be that of the virtual promastigote, and show sample kinematics in Fig. C1 for a single initial configuration. Fig. C1 a presents the motion of swimmers when a repulsive boundary force is included. We observe that, despite differing in body length scale, the swimmers follow qualitatively-similar paths in phase space and exhibit the same overall behaviours. The larger-bodied swimmers are seen to undergo reduced reorientation and displacement in phase space when compared with smaller-bodied virtual swimmers, consistent with larger bodies having increased overall drag as well as increased near-wall hydrodynamic resistance, the latter as presented in Table 1. Without repulsive short range surface forces the same level of qualitative agreement is present, as evident from the time series of Fig. C1b, with increased reorientation towards the boundary for larger-bodied swimmers.

Similar consideration of variation in body aspect ratio from that of the virtual promastigote yields qualitatively-unchanged dynamics, where additionally we observe that decreases in aspect ratio result in reduced swimmer velocities and correspondingly shortened trajectories in phase space for a given simulation time. With the virtual promastigote previously having had an aspect ratio of  ∼ 3.14, here the aspect ratio was sampled from the range 2–5, capturing the typical variation seen in *Leishmania* promastigotes (Wheeler et al., 2011). Hence we have seen that the reported behaviours of virtual promastigotes are robust to observed variations in both aspect ratio and body length scale.

## Appendix D Supplementary materials

Supplementary Data S1 Supplementary Raw Research Data. This is open data under the CC BY license http://creativecommons.org/licenses/by/4.0/ Supplementary Data S1

Supplementary Movie 1 Supplementary Raw Research Data. This is open data under the CC BY license http://creativecommons.org/licenses/by/4.0/ Supplementary Data S2

Supplementary Movie 2 Supplementary Raw Research Data. This is open data under the CC BY license http://creativecommons.org/licenses/by/4.0/ Supplementary Data S3

Supplementary Movie 3 Supplementary Raw Research Data. This is open data under the CC BY license http://creativecommons.org/licenses/by/4.0/ Supplementary Data S4

Supplementary Movie 4 Supplementary Raw Research Data. This is open data under the CC BY license http://creativecommons.org/licenses/by/4.0/ Supplementary Data S5

Supplementary Movie 5 Supplementary Raw Research Data. This is open data under the CC BY license http://creativecommons.org/licenses/by/4.0/ Supplementary Data S6
