# Supplementary material for: Boundary behaviours of Leishmania mexicana: A hydrodynamic simulation study
Source: J Theor Biol. 2019 Feb 7;462:311–20. doi: 10.1016/j.jtbi.2018.11.016 (PMC6333917; doi:10.1016/j.jtbi.2018.11.016)
Supplement: Supplementary Data S1 — Supplementary Raw Research Data. This is open data under the CC BY license http://creativecommons.org/licenses/by/4.0/ [file mmc1.pdf]

Supplementary Material for the manuscript entitled ‘Boundary behaviours of Leishmania mexicana: a hydrodynamic simulation study’ by Benjamin J. Walker, Richard J. Wheeler, Kenta Ishimoto and Eamonn A. Gaffney.

- Supplementary Movie 1 - The model tip-to-base flagellar beating of a virtual promastigote.
- Supplementary Movie 2 - A virtual promastigote on a collision-bound swimming trajectory near a no-slip wall.
- Supplementary Movie 3 - The deflection of an incoming virtual promastigote away from a planar boundary with additional surface repulsion.
- Supplementary Movie 4 - The swimming of a virtual promastigote close to a planar boundary with additional surface repulsion.
- Supplementary Movie 5 - The swimming of a virtual pusher with the morphology of the virtual promastigote close to a planar boundary with additional surface repulsion.
